# Cutting-edge assays for mirabegron and tadalafil combo therapy for benign prostatic hyperplasia; insilico kinetics approach; multi trait sustainability assessment

**DOI:** 10.1186/s13065-025-01497-z

**Published:** 2025-05-23

**Authors:** Sara I. Aboras, Mohamed R. Abdelhakim, Hadir M. Maher, Rasha M. Youssef

**Affiliations:** 1 Pharmaceutical Analytical Chemistry Department, Faculty of Pharmacy, University of Alexandria, Elmessalah, Al-mesallah, Alexandria, 21521 Egypt; 2 Alexandria’s Main University Hospital, University of Alexandria, Alexandria, Egypt; 3 Faculty of Pharmacy, Alamein International University, Alamein, Matrouh Egypt

**Keywords:** Mirabegron, Tadalafil, HPLC, Spectrophotometry, Benign prostatic hyperplasia, Sustainability

## Abstract

The combination therapy of mirabegron (MIR) and tadalafil (TAD) has gained significant attention in clinical practice for the treatment of Benign Prostatic Hyperplasia as well as overactive bladder syndrome. This study demonstrates the development and validation of two analytical methods for the concurrent determination of tadalafil and mirabegron in their synergistic combo therapy. Spectrophotometry and stability-indicating HPLC were employed for the accurate quantification of both drugs. The spectrophotometric methods were based on derivative, Fourier and ratio derivative of the two drugs, providing simpler, rapid alternatives for routine analysis. The stability-indicating HPLC method was developed using a reverse-phase column with a methanol-phosphate buffer gradient mobile phase, achieving optimal separation with detection at 250 nm for MIR and 225 nm for TAD. Forced degradation studies were performed under acidic, basic, oxidative, neutral, thermal, and photolytic conditions, confirming the method’s ability to quantify MIR and TAD in presence of degradation products and establish drug stability. Both techniques were validated based on ICH guidelines, demonstrating excellent linearity, precision, accuracy, and sensitivity. The methods were successfully applied to the analysis of bulk powder and in dosage forms. Both methods were evaluated and found to be environmentally friendly, receiving approval as “green” according to the AGREE (Assessment Tool for Greenness) method, “white” under the RGB12 criteria, and “blue” according to the BAGI (Blue Analytical Greenness Index). These evaluations demonstrate the sustainability and eco-friendliness of the proposed analytical techniques. Furthermore, artificial intelligence was employed through an online tool to ensure that there were no potential drug-drug interactions between MIR and TAD, offering an additional layer of safety in clinical applications. This research contributes to the enhancement of quality control for patients undergoing combination therapy with MIR and TAD and stability testing.

**Graphical Abstract:**

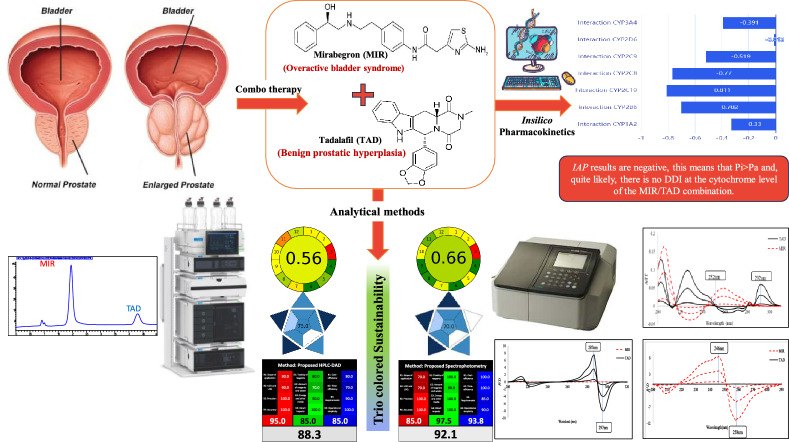

**Supplementary Information:**

The online version contains supplementary material available at 10.1186/s13065-025-01497-z.

## Introduction

Benign prostatic hyperplasia (BPH) as well as overactive bladder syndrome (OAB) are extremely common disorders that often result in lower urinary tract symptoms (LUTS) and a decreased quality of life, especially in the elderly [1].

Based on autopsy studies, the age-specific prevalence of BPH is estimated to be 8% in the fourth decade, 50% in the sixth, and 80% in the ninth decade of life [2]. On the other hand, it has been observed that OAB symptoms in men are frequently triggered by a bladder outlet obstruction brought on by BPH and they are treated accordingly [3].

For BPH, phosphodiesterase type 5 inhibitors, or PDE5-Is, are the first-line of treatment through relaxation of smooth muscles, increasing pelvic blood perfusion, and probably modifying afferent nerve activity. Several countries have legalized the use of tadalafil (TAD), a PDE5-I, for the treatment of LUTS/BPH, and prior randomized studies have demonstrated that its efficacy is comparable to that of α1-blockers [4]. Although TAD may alleviate OAB and other voiding and storage symptoms, OAB symptoms could still persist in some cases. Thus, several combination treatments or add-on therapies have been revealed to be beneficial for these patients. Anticholinergics were the most commonly utilized combination, and treating OAB symptoms brought on by BPH with these medications can be helpful [5]. However, adding anticholinergics may have a number of negative consequences, including dry mouth, constipation, impaired vision, and a loss in cognitive function and hence, alternative therapeutic approaches need to be thought of. As a result, Mirabegron (MIR), a β3-adrenoceptor (AR) agonist, has been found to be as effective as anticholinergics in treating OAB associated with BPH. However, it has fewer side effects, such as constipation and dry mouth, and is more affordable and palatable [4, 5].

**Fig. 1:**
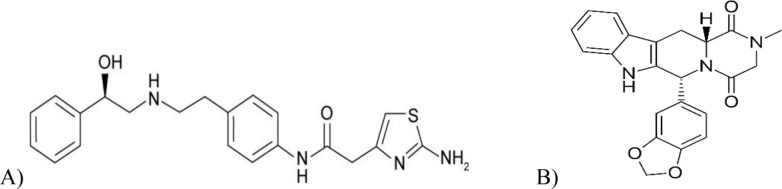
Chemical structures of **A** Mirabegron and **B** Tadalafil

Recent studies have proved that combo therapy or co-administration of TAD with MIR was efficient, and its safety was found to be on par with TAD mono-treatment in patients with BPH suffering from persistent storage symptoms [6]. Therefore, TAD plus MIR, Fig. 1, is promising effective treatment option, and it can enhance quality of life in BPH patients. Furthermore, among individuals with OAB brought on by BPH, the efficacy of TAD/MIR combo therapy seemed to be greater than that of TAD monotherapy and is linked to a decreased incidence of LUTS [6, 7].

For figuring out a combo therapy’s expiration date and storage requirements, stability studies are essential. Patient safety and product quality are impacted by their analysis and testing of crucial quality factors. It is advised to conduct stress testing to concentrate on important quality factors, such as exposure to acid/base, humidity, temperature, oxidizers, and transportation extremes. Chromatographic methods are preferred in stability investigations because they can separate analytes completely in presence of degradation products in reasonable run times [8, 9].

For the determination of MIR, the literature reviews had shown many analytical techniques in bulk, pharmaceutical dosage forms, and biological fluids, alone or with other drugs, including spectrophotometric [10, 11], spectrofluorometric [12, 13], LC–MS/MS [14–16], LC-DAD [17], HPTLC [18], and electrochemical approaches [19].

Numerous analytical techniques analyzed TAD either alone or together with other drugs, such as spectrophotometric [20–22], spectrofluorimetric [23, 24],.voltametric [25], HPTLC [26, 27], LC-DAD [22, 28], LC-FLD [28], and LC–MS/MS [29, 30].

Literature review reveals no reports regarding the concurrent determination of MIR and TAD. Consequently, the invention and validation of spectrophotometric and stability-indicating chromatographic techniques for the concurrent determination of TAD and MIR for quality control purposes will be the main emphasis of this study.

While the spectrophotometric techniques comprised derivative spectra of absorption curves (D), ratio derivative (RD) and Fourier functions convolution of absorption spectra (FF), the chromatographic assay utilized gradient elution for separating this combo therapy. The referenced drugs' mixes in dosage forms were effectively determined using the suggested methods.

Additionally, different greenness, whiteness and blueness assessment tools were used to compare and assess the methods’ sustainability. Moreover, *insilico* approach was utilized to evaluate any potential drug-drug interactions (DDIs) between TAD and MIR to ensure both safety and efficacy as the cited papers mentioned their synergistic effect at the pharmacodynamics level [6, 7].

## Experimental

### Materials and reagents

MIR reference standard was purchased from Sigma-Aldrich^®^ (Purity 99.6%). TAD reference standard was provided by *Al-Andalous* Pharma, Cairo, Egypt (Purity 98.2%). The commercial products, Bladogra^®^ (by APEX pharma, New Cairo, Egypt) containing 25 mg MIR and Diamonrecta^®^ (by EVA PHARMA, Giza, Egypt) containing 20 mg TAD were purchased from the Egyptian market. HPLC-grade methanol (MeOH) as well as acetonitrile (ACN) were obtained from Baker, Ireland. Triethyl amine (TEA) was purchased from Sigma-Aldrich^®^. Hydrochloric acid (HCl) and sodium hydroxide (NaOH) of analytical quality were supplied by El-Nasr Chemical Industry Company in Egypt. Additionally, 20% hydrogen peroxide (H_2_O_2_) was also acquired from El-Nasr Chemical Industry Company. Distilled water was obtained via simple distillation using water distillator.

### Apparatus

#### Spectrophotometer

A Shimadzu UV-1800 PC, Kyoto, Japan spectrophotometer (UV–Vis double beam) utilized to do the spectrophotometric measurements and was outfitted with matched quartz cells with corresponding 1 cm path lengths.

#### HPLC

A computer running Agilent ChemStation software was linked to the HPLC–DAD system, which included an Agilent 1260 series (Agilent Technologies, Santa Clara, CA, USA) with multiple wavelength diode array detectors G1315C/D and G1365C/D, an automated injector, a quaternary pump, and a vacuum degasser. The Agilent Eclipse Plus C_18_ (4.6 × 100 mm × 3.5 µm) column was utilized.

### Stock solutions

Stock solutions were prepared in HPLC-grade MeOH to get a 1000 µg/mL of MIR and TAD. The prepared MIR and TAD stock solutions were kept at 4°C in refrigerator for almost one week.

### General procedure

#### Construction of calibration curves

##### Spectrophotometry

Different portions of the methanolic stock solutions (1000µg/mL) for each drug were moved to a set of 10 mL-volumetric flasks to prepare MIR/TAD calibration sets in the range 1–20 µg/mL for both drugs then completed to their marks with distilled water.

##### HPLC

For chromatographic analysis, linearity solutions were prepared in 10 mL-volumetric flasks by accurately transferring set volumes of stock solutions for each drug and then completing to the mark with the starting ratio of the mobile phase to get a series of MIR and TAD concentrations in the range 0.85–100 µg/mL for MIR and 0.65–100 µg/mL for TAD.

### Synthetic mixtures

For spectrophotometry, accurate volumes of stock solutions were moved to a set of 10 mL-volumetric flasks and then completed to the mark with distilled water to get mixtures of the following concentrations 20, 2 µg/mL, 10, 10 µg/mL and 3, 15 µg/mL for MIR and TAD, respectively. Similarly, the same procedure was done with the chromatographic method but the final dilution was performed using the same diluting solvent or the starting ratio of mobile phase used for construction of calibration curves.

### Analysis of dosage forms

For both spectrophotometry and HPLC, ten tablets of each Bladogra^®^ and Diamorecta^®^ were weighted, crushed, and the corresponding weight of 50 mg MIR and 5 mg TAD were underwent a rigorous homogenization process using mechanical mixing to ensure uniform analyte distribution. Then this mix was transferred in 50-mL volumetric flask. Around 30 mL MeOH was then transferred to extract both drugs by sonication for 20 min followed by completing the volume to the mark with MeOH. The filtration protocol was carefully optimized to ensure complete removal of insoluble excipients from the dosage form. A 0.45 µm PTFE membrane filter was selected based on its chemical compatibility and ability to effectively remove particulate matter without affecting analyte recovery. To confirm the absence of significant analyte adsorption onto the filter, a recovery study was conducted. Known concentrations of MIR and TAD were filtered through the selected membrane, and the drug content was measured before and after filtration. The recovery rates were found to be 98.7% for MIR and 99.2% for TAD, indicating negligible adsorption. These results demonstrate that the filtration protocol is both effective and reliable for dosage form analysis. After filtration of the tablets extract, accurate volume of the extract was diluted to get 20 and 2 µg/mL for MIR and TAD, respectively to be analyzed using the proposed methods.

### Experimental conditions

#### Spectrophotometry

##### *First derivative spectrophotometry (*^*1*^*D)*

The first derivative spectra ^1^D (with ∆λ of 2 nm) of MIR and TAD standard solutions and the synthetic mixtures were recorded vs blank. For MIR, there was no point of zero crossing for TAD to analyze MIR. However, the ^1^D at 294 nm (zero-crossing point for MIR) was measured then plotted against the concentrations of TAD to get its calibration graph.

##### Fourier function convolution spectrophotometry (A/FF)

This Fourier function convolution spectrophotometry (A/FF) depends on convolution of the zero-order UV spectra of MIR and TAD standards and their synthetic mixtures by combined trigonometric Fourier functions using the following equation for 8 consecutive wavelengths measured at 1 nm interval:

1 $${t{\prime}}_{r}= [( + 1.707){Ar}_{0}+ ( + 0.707){Ar}_{1} + ( - 0.707){Ar}_{2} +(- 1.707){Ar}_{3} + ( - 1.707){Ar}_{4} + ( - 0.707){Ar}_{5} +( + 0.707){Ar}_{6} + ( + 1.707){Ar}_{7}]/4$$

where t’_r_ is the Fourier function coefficient and eight zero-order absorption values are represented by Ar0-Ar7. The specified combined FF, which were computed as follows, are shown by the numbers in parenthesis:

T´ = [cos x + cos (x + 45)] (2).

FF coefficients were recoded at 252 nm (TAD zero crossing) to determine MIR. Similarly, the coefficients were also recorded at 292 nm (zero crossing of MIR) and were used for the determination of TAD. By connecting the FF coefficients determined at the chosen sites to the relevant component concentration, calibration curves as well as the regression equations produced from them were created.

##### *Ratio derivative spectrophotometry (R*^*1*^*D)*

To obtain MIR concentration, the absorption spectra of MIR/TAD mixtures were divided by those of standard TAD (1 μg/mL). The R^1^D was then recorded to determine MIR concentrations at 246 and 258 nm (peak to peak). Similarly, for the same MIR/TAD mixtures, were divided by that of standard MIR (10 μg/mL). The R^1^D of the ratio spectra was then recorded to determine TAD concentrations at 287 and 297 nm (peak to peak).

#### Chromatographic conditions

An Agilent Eclipse Plus -C_18_ analytical column (100 × 4.6 mm, 3.5 µm) was used for the separation process. For the first three min, the gradient mobile phase system was set up with 40: 60% (v/v), solvent A (phosphate buffer 10 mM, pH 7 with triethylamine, 1% (v/v)): solvent B (MeOH) followed by step change to 20:80, v/v, respectively for two min. At 5 min, the system returned to the initial ratio of the mobile phase to permit column equilibration before the next injection. A membrane filter with a pore size of 0.45 µm was used to filter the buffer, and 25 ºC was the setting for the temperature during the run. Using DAD, the detection wavelength was tuned at 250 nm for MIR and 225 nm for TAD. With an injection volume 10-µL and a flow rate 1 mL/min, the run duration was 6 min in total. The 10 µL injection volume was selected after evaluating different volumes (5–20 µL) to balance sensitivity, peak shape, and solvent consumption. Ten µL volume led to reduce solvent consumption while maintaining peak integrity. Volumes lower than 10 µL led to lower response with peak distortion. A combination 80:20 MeOH: Water in a volume of 100 µL as washing solution of the needle between injections effectively dissolves TAD (highly organic-soluble) and the water-soluble MIR and thus eliminated carry over effect.

### Forced degradation and stability studies

Forced degradation tests were performed on MIR and TAD separately and in mixture using working solutions (500 µg/mL for each) with an amount of 1 mL. After degradation, the solutions were neutralized, if needed, followed by completing with distilled water to the full level in 10-mL volumetric flasks series. A final concentration of 50 µg/mL was achieved for both MIR and TAD. Each degradation condition was done in replicates.

#### Acidic and basic hydrolysis

For acidic and basic hydrolysis, respectively, 1 mL volumes of 1 M HCl or 1 M NaOH were added to the MIR and TAD solutions. After that, the solutions were heated in a thermostatic water bath at 90 °C for one hour for both basic and acidic hydrolysis. After the specific time, the solutions have been cooled and then neutralized to pH 7 at the predetermined intervals. Degraded solutions were diluted with distilled water in 10-mL volumetric flasks.

#### Neutral hydrolysis

1 mL of distilled water was added to the MIR and TAD solutions. The mixture was heated up in a water bath at 90 ℃ for 1 h, then cooled and completed to the volume and concentration specified above.

#### Oxidative degradation

MIR and TAD solutions were treated with 1 mL of hydrogen peroxide (6% v/v) and kept at room temperature for 1 h. The solutions were then diluted as described above.

#### Photolytic degradation

Photo-stability was conducted by exposing MIR and TAD stock solutions (1 mL, 500 µg/mL) to light for 24 h. Following the mentioned period, the volume was diluted, as illustrated above.

#### Dry heat

In a controlled-temperature oven, a precise weight of 10 mg of the powdered MIR and TAD, both alone and in combination, were maintained for one hours at 90 ± 1°C. Following the specified amount of time, the treated powder was dissolved in methanol in a 10-mL volumetric flask to form a 500-μg/mL stock solution. One mL was then further diluted 10 times using distilled water before being added to the HPLC system.

## Results and discussion

The combination of MIR and TAD combo therapy demonstrated a synergistic strategy in the treatment of LUTS/BPH and LUTS/OAB. For quality control purposes, simultaneous determination of the two medicines is crucial. Different analytical techniques were suggested for the concurrent determination of MIR and TAD: spectrophotometric based methods, A/FF, R^1^D, and an HPLC method. On the other hand, one UV spectrophotometric technique, ^1^D, was proposed for TAD analysis alone. To extend the applicability of our HPLC method to stability-indicating property and to ensure safe combinations of these medications, a stress study was performed and succeeded in separating the drugs from degradation products.

### Method optimization

#### Spectrophotometric optimization

**Fig. 2:**
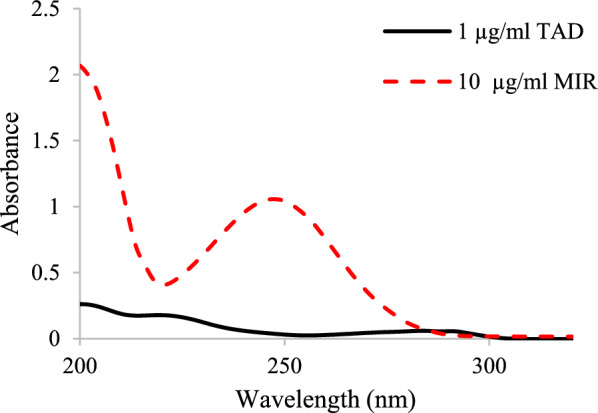
zero order spectra of MIR & TAD at their therapeutic dosage ratio (10:1)

The MIR and TAD absorption spectra revealed great overlap during their determination in the reported therapeutic dose of 10:1 for MIR and TAD, respectively, Fig. 2. Thus, the concurrent determination of the two drugs was difficult using direct absorption spectra. Accordingly, a variety of mathematical techniques for manipulating absorbance data were tested in order to achieve spectral resolution without requiring pre-separation steps. Three methods were proposed, the first one depended on derivative spectra of absorption curves, the second used trigonometric FF for spectral convolution, and the last used ratio spectra of derivative absorption curves.

It is important to highlight that water, EtOH, and MeOH were examined as diluting solvents. Water was the ideal solvent for the analytical and environmentally friendly requirements in proposed methods. MeOH and EtOH produced similar intensities for both drugs but with less ecofriendly impact, Fig.S2.

##### *First derivative method (*^*1*^*D)*

By utilizing Excel software to differentiate absorbance data with respect to λ, ^1^D (dA/dλ) signals could be generated. Generally, these signals have distinct maxima or minima and could be used to determine single analytes—by crossing zero points.

**Fig. 3:**
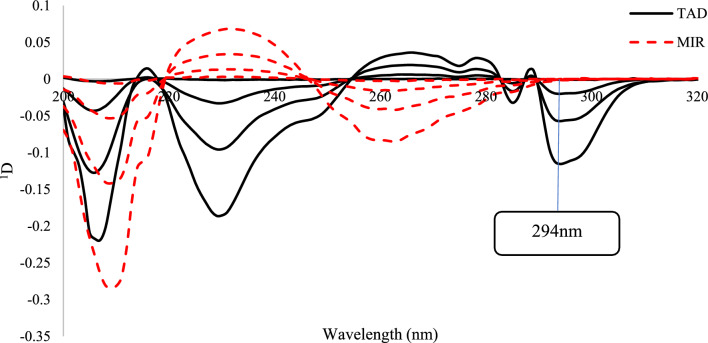
Overlaid of ^1^D plots for MIR & TAD at four concentrations (1,5, 10 and 20 µg/mL) for each component

The effect of wavelength interval, ∆λ, was investigated in order to achieve the highest sensitive ^1^D signals with least noise amount. It was determined that a 2 nm wavelength interval was suitable. The process of differentiating zero order spectra produced ^1^D, with a peak of TAD at 294 nm with a zero crossing of MIR, Fig. 3. The direct determination of MIR using ^1^D method was not achievable in the presence of TAD.

##### Fourier function convolution spectrophotometry (A/FF)

**Fig. 4:**
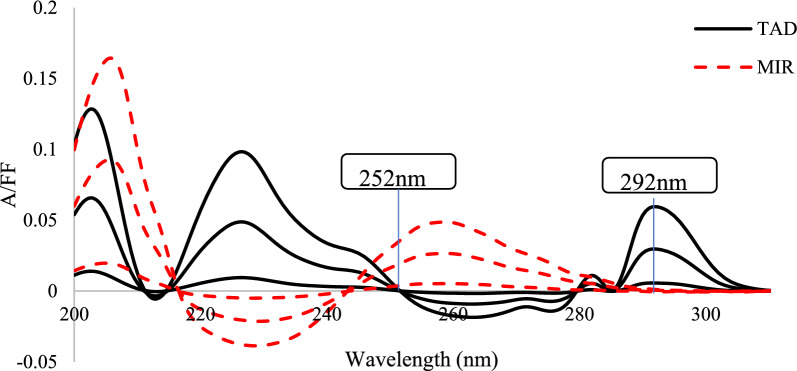
Overlaid of A/FF plots for MIR & TAD at three concentrations (1,5, and 10 µg/mL) for each component

First, eight points trigonometric FF, with a 1 nm interval, were used to convolute both MIR and TAD spectra using excel software. Displaying the computed coefficients as a function of λ resulted in convoluted spectra, Fig. 4. This figure showed that MIR could be determined at 252 nm, when TAD's contribution was nil while TAD could be computed at 292 nm, a zero MIR contribution.

Calibration curves as well as the regression equations were computed by relating the FF coefficients, calculated at the selected points, to the respective component concentrations.

Applying FF convolution to various spectral data types is very helpful in removing various interferences and eliminating spectral overlap [31, 32]. Thus, Fourier functions could potentially be employed as a more accurate substitute for derivative spectrophotometry. Even at low concentrations, Fourier function convolution may be quite helpful in solving binary mixes with substantially overlapping spectra because of its ability to remove various interferences. High levels of selectivity for a certain drug with minimal interference from other compounds are guaranteed by choosing the function type, quantity of data points, and wavelength interval optimally.

##### *Ratio derivative spectrophotometry (R*.^*1*^*D)*

To get ratio spectra, absorption spectra were divided either by 1 μg/mL TAD, which was used for the selective measurement of MIR, or by 10 μg/mL MIR, which was used for the selective determination of TAD.

The magnitude of the ratio spectra is mostly determined by the divisor concentration, which is why several concentrations of TAD (1, 5, and 10 μg/mL) and MIR (1, 5, 10, and 15 μg/mL) were investigated. The concentrations of the divisors, 1 μg/mL TAD and 10 μg/mL MIR, were chosen according to the lowest noise level and the highest sensitivity.

**Fig. 5:**
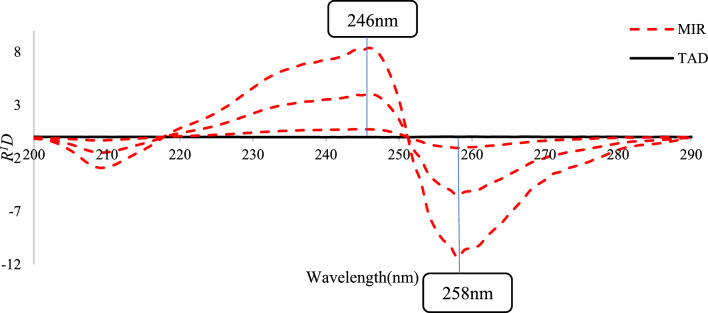
Overlaid *R*^*1*^*D* spectra of MIR at 1, 5, 10 μg/mL with synthetic mixtures of different ratios divided by 1 µg/mL TAD

**Fig. 6:**
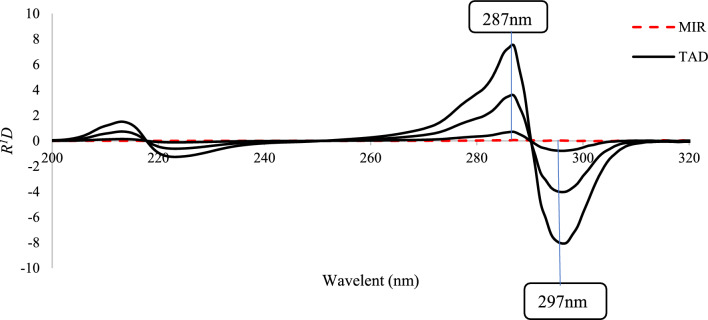
Overlaid* R*^*1*^*D* spectra of TAD at1, 5, 10 μg/mL TAD with synthetic mixtures of different ratios divided by 10 µg/mL MIR

Plotting the estimated ratio derivative spectra versus wavelength resulted in the appropriate convoluted curves, Figs. 5 and 6. Figure 5 made it evident that the coefficients chosen at peak-to-peak measurements of 246 and 258 nm could be used to determine the concentration of MIR whereas Fig. 6 showed that the coefficients at peak-to-peak measurements of λ 287 and 297 nm were corresponding to the concentration of TAD.

This approach used points of coincidence across the spectra of the binary mixture and the standard solution of only one compound with the same concentration to determine the working wavelengths, Fig. S1. This makes peak-to-peak results feasible, which raises the analytical signals' sensitivity.

#### Chromatographic optimization

The goal of the suggested HPLC technique was to create a valid chromatographic system that could separate and elute MIR and TAD simultaneously. The following experimental parameters were adjusted to optimize the analytical process's sensitivity and selectivity.

##### Stationary phase

A variety of stationary phases were tested. Both MIR and TAD peaks were eluted lately using C_8_ and C_18_ (5 µm, 250 × 4.6 mm) columns, whereas they were successfully separated within short run time using a C_18_ (3.5 µm, 100 × 4.6 mm) column.

##### Organic modifier

The peak form of both medications under investigation was significantly influenced by the type of organic modifier. A variety of organic solvents, including ACN, MeOH, and EtOH, had been explored. Peak broadening, reduced efficiency, and shape distortion of the MIR and TAD peaks were the results of using EtOH in the mobile phase. The use of ACN caused MIR to be eluted relatively early, causing its peak to be overlapped with the solvent front. However, the elution of MIR and TAD with a suitable peak shape for both and retention time for MIR was made possible by the use of MeOH.

The mobile phase containing varying amounts of MeOH (0%, 25%, 50%, 60%, and 80%) was used to inject binary drug combinations, Table S1. Initially, 60% MeOH was chosen for separation resulting in a reasonable elution of MIR (2 min) but late TAD elution (10 min) with high tailing factor. Higher ratio of MeOH caused rapid elution of MIR near the solvent front. Trying ACN instead of MeOH resulted in a very rapid elution of MIR with solvent front even though reasonable elution of TAD had been achieved. So, gradient elution was eventually used with 60% MeOH for the first three min, then the percentage was increased to 80% for 2 min to achieve optimum separation with reasonable retention times for both peaks, Table S1. At 5 min, the system returned to the initial ratio of the mobile phase to permit column equilibration before the next injection.

##### pH of the aqueous phase

The impact of the eluent's aqueous component's pH was examined. A range of pH values (2.0–7.0) with 10 mM phosphate buffer (modified by sodium hydroxide or orthophosphoric acid), in conjunction with MeOH in the previously mentioned gradient system, were tested.

The best resolution of the cited drugs was ideally achieved using pH of 7 matching the pKa values of both drugs; MIR 13.84, 9.62 and TAD 15.17, -4.2, from drug bank. Additionally, TEA was incorporated to the aqueous portion as a competitive base to TAD and thus improved peak tailing.

##### Detection wavelength

Various wavelengths were tested for the quantification of the suggested drugs for the sake of improving the method's sensitivity. The best sensitivity was gained with detection at 250 nm for MIR and 225 nm for TAD.

**Fig. 7:**
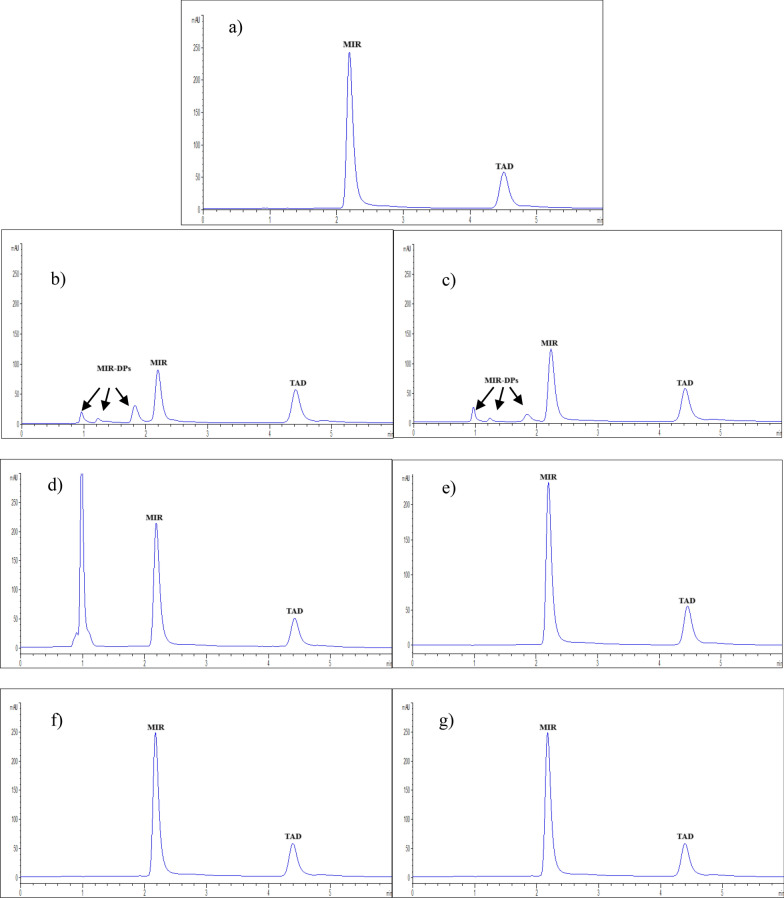
HPLC chromatograms of 50 µg/mL of MIR and TAD **a** without degradation, using **b** alkaline, **c** acidic, **d** peroxide, **e** water, **f** light and **g** dry heat stress conditions

Peak sharpness and resolution within acceptable runtime, Fig. 7a, demonstrates the notable separation of MIR and TAD. Table 1 demonstrates that the system suitability parameters of the chromatographic separation of our binary method meets the FDA guidelines [33].

**Table 1:** System suitability parameters for the HPLC–DAD determination of MIR and TAD^a^

	MIR (250 nm)	TAD (225 nm)
t_R_ ± SD (min)^b^	2.21 min ± 0.01	4.52 min ± 0.01
Plates (N)	2181	2505
Resolution^c^	8.09	
Capacity factor (k')	1.12	3.67
Tailing Factor	1.05	0.98
Selectivity factor (α)	3.28	

^a^System suitability recommendations: k' (1–10), N > 2000, α > 1, R_s_ > 2 and T_f_ (0.8 -1.2)[39]

^b^Average t_R_ ± SD of three determinations

^c^Resolution between MIR and TAD

### Methods validation

Validation of our suggested methods was done in consistent with ICH standards [34] as follows:

#### Linearity, limit of detection (LOD) and limit of quantitation (LOQ)

The calculated concentrations of MIR and TAD were related to the computed values for A/FF, R^1^D or HPLC area for both drugs, as well as ^1^D for TAD only, each of which was chosen at the previously set wavelength and chromatographic conditions. Regression coefficient values (r) for each of the recommended methods were higher than 0.999, suggesting a boosted degree of linearity as shown in Table 2. Moreover, the linearity range in HPLC method was wider than spectrophotometric ones, Table 2, as the linearity range in spectrophotometer is limited to a narrow absorbance range, 0.3–0.9. LOD and LOQ of MIR and TAD were determined as stated in ICH recommendations [34], Table 2. HPLC minimizes background noise by separating interfering substances before detection, leading to better signal clarity. Thus, LOD and LOQ of HPLC method were about 100 times lower than those of spectrophotometric methods, Table 2.

**Table 2:** Analytical performance data of spectrophotometric and HPLC methods for MIR and TAD

Analyte	MIR			TAD			
Method	A/FF	R^1^D	HPLC	^1^D	A/FF	R^1^D	HPLC
Wavelength (nm)	252	246 & 258	250	294	292	287&297	225
Linearity Range (µg/mL)	1–20		0.85–100	1–20			0.65–100
LOD (µg/mL)	3.18 $\times {10}^{-1}$	2.83 $\times {10}^{-1}$	1.31 $\times {10}^{-3}$	3.24 $\times {10}^{-1}$	2.95 $\times {10}^{-1}$	2.65 $\times {10}^{-1}$	3.41 $\times {10}^{-3}$
LOQ (µg/mL)	9.64 $\times {10}^{-1}$	8.59 $\times {10}^{-1}$	3.97 $\times {10}^{-3}$	9.83 $\times {10}^{-1}$	8.93 $\times {10}^{-1}$	8.04 $\times {10}^{-1}$	1.13 $\times {10}^{-2}$
Correlation coefficient	0.9993	0.9994	0.9996	0.9996	0.9997	0.9998	0.9996
Slope	3.77 $\times {10}^{-3}$	1.83	3.11 $\times {10}^{1}$	4.78 $\times {10}^{-3}$	2.72 $\times {10}^{-3}$	1.78	5.49 $\times {10}^{1}$
SE of slope	6.00 $\times {10}^{-5}$	2.75 $\times {10}^{-2}$	6.67 $\times {10}^{-1}$	7.00 $\times {10}^{-5}$	3.00 $\times {10}^{-5}$	1.68 $\times {10}^{-2}$	1.93
Intercept	2.00 $\times {10}^{-5}$	-2.32 $\times {10}^{-1}$	1.18 $\times {10}^{1}$	-1.44 $\times {10}^{-3}$	-1.08 $\times {10}^{-3}$	-4.91 $\times {10}^{-1}$	-3.41
SE of intercept	3.60 $\times {10}^{-4}$	1.57 $\times {10}^{-1}$	1.63 $\times {10}^{1}$	4.70 $\times {10}^{-4}$	2.40 $\times {10}^{-4}$	1.43 $\times {10}^{-1}$	6.42

^a^average of three determinations

#### Accuracy and precision

The assay of MIR and TAD synthetic combinations, covering the linearity range with various ratios confirmed the validity of the suggested procedures. Good mean % recoveries (100 ± 2%) along with optimum % RSD (not exceed 2%), Table S2, indicated excellent accuracy alongside with precision of the proposed methods for all mixture ratios, including the challenging ratio of the dosage form 10:1.

#### Selectivity

Selectivity was evaluated in MIR and TAD using tablets’ extract, Table 3, to ensure absence of interferences from excipients in the tablets. Both spectrophotometric and chromatographic methods showed superior percentage mean recoveries (100% ± 2) for both MIR and TAD, with relative SD values (± 2%) in all different ratios. Additionally, in HPLC, DAD was used to determine peak purity, which guarantees MIR and TAD peaks purity, Fig. S3. The above-mentioned results guaranteed methods’ selectivity.

**Table 3:** Assay of MIR and TAD in laboratory prepared tablets using the proposed methods

	MIR			TAD			
Method	A/FF	R^1^D	HPLC	^1^D	A/FF	R^1^D	HPLC
Mean $\pm$ SD	99.55 $\pm$ 1.25	100.22 $\pm$ 0.93	99.20 $\pm$ 0.99	99.97 $\pm$ 0.98	100.95 $\pm$ 1.35	99.56 $\pm$ 1.20	101.12 $\pm$ 0.96
Er%	− 0.45	0.23	− 0.80	− 0.03	0.95	− 0.44	1.12
F calculated	1.43			2.66			
F critical	3.68			3.10			

#### Robustness of the HPLC method

By assessing MIR and TAD at the same concentration levels as previously mentioned but with other parameters (temperature, wavelength and pH) that were slightly altered, as shown in table S3, the robustness of the suggested approaches was evaluated.

It was discovered that minor intentional adjustments to the parameters had no discernible impact on the assay of MIR and TAD. Satisfactory robustness shown by nearly unchanged responses (peak areas 100 ± 2%), and/or retention times (SD ≤ 2%) values of both MIR and TAD in the HPLC method.

### Analysis of dosage forms

The suggested techniques were applied for quantification of both drugs in their dosage forms. The concentrations of MIR and TAD were calculated from their corresponding regression equations. Single factor: One-way ANOVA [35] was used for statistical comparison, Table 3. There was no discernible difference between the approaches because the computed F values of 1.43 for MIR, and 2.66 for TAD did not surpass the critical values of 3.68 and 3.10, respectively.

### Stability-indicating study

The stability-indicating capability of the HPLC method was evaluated by subjecting MIR and TAD to various forced degradation conditions, including oxidative, thermal, photolytic, neutral, acidic, and alkaline stress, in accordance with ICH Q1A(R2) guidelines [36]. The results demonstrated that MIR and TAD remained stable under oxidative, thermal, light, and neutral conditions, as no significant degradation products were observed, and peak purity remained intact, Figs, 7 and Table S4. However, under acidic and alkaline hydrolysis, TAD exhibited remarkable stability, while MIR underwent 58.5% and 47.2% degradation, respectively, forming additional peaks at 1, 1.2 and 1.8 min. corresponding to degradation products (DPs), Figs, 7. The peak purity analysis confirmed that the method effectively separated DPs from the parent MIR, validating its stability-indicating nature, Table S4. The degradation of MIR under acidic and alkaline conditions was most likely attributed to the hydrolysis of the amide group and thus suggests its susceptibility to acidic and alkaline breakdown, emphasizing the need for controlled pH conditions in formulations and storage. However, the degradation patterns of the medications as a mixture and as singles did not differ, suggesting that these drugs could be safely used together. Moreover, the obtained stability results were in accordance with previously reported literature [18, 37]. These findings confirm that the developed HPLC method is selective, reliable, and suitable for stability studies, ensuring accurate quality control of MIR-TAD combination therapy.

### Trio colored assessment, greenness, blueness and whiteness, of the proposed analytical methods

Environmental methods have recently drawn more attention in a variety of analytical techniques, such as chromatography [9, 38], and spectroscopy [39–41]. Additionally, the three-color-coded methodology provides a comprehensive evaluation of every analytical step [42]. The concerns of environmental safety as well as sustainable development objectives must be addressed by the analytical procedure.

#### Greenness assessment

Based on the twelve green analytical chemistry (GAC) foundations, AGREE [43] which is a free greenness evaluation tool, was released in June 2020. A fraction of one represents the overall score in AGREE, which goes from zero (lowest green) to one (highest green). The twelve parts that make up the automatically created pictogram each have a unique fundamental hue that varies from deep green to deep red depending on how well it matches the greenness. Our proposed procedures received eco-friendliness grades of 0.66 and 0.56 for spectrophotometric and chromatographic methods, respectively, out of 1 from the AGREE tool, Table 4. The difference in the scores between the two methods is attributed to the used diluting solvent, water, which is greener than MeOH, the organic mobile phase in HPLC method. Moreover, lower energy consumption along with higher sample numbers contributed to the increased spectrophotometry AGREE value over HPLC. Thus, the suggested approaches closely complied with its greenness attribute, according to the results, Table 4.

**Table 4:**
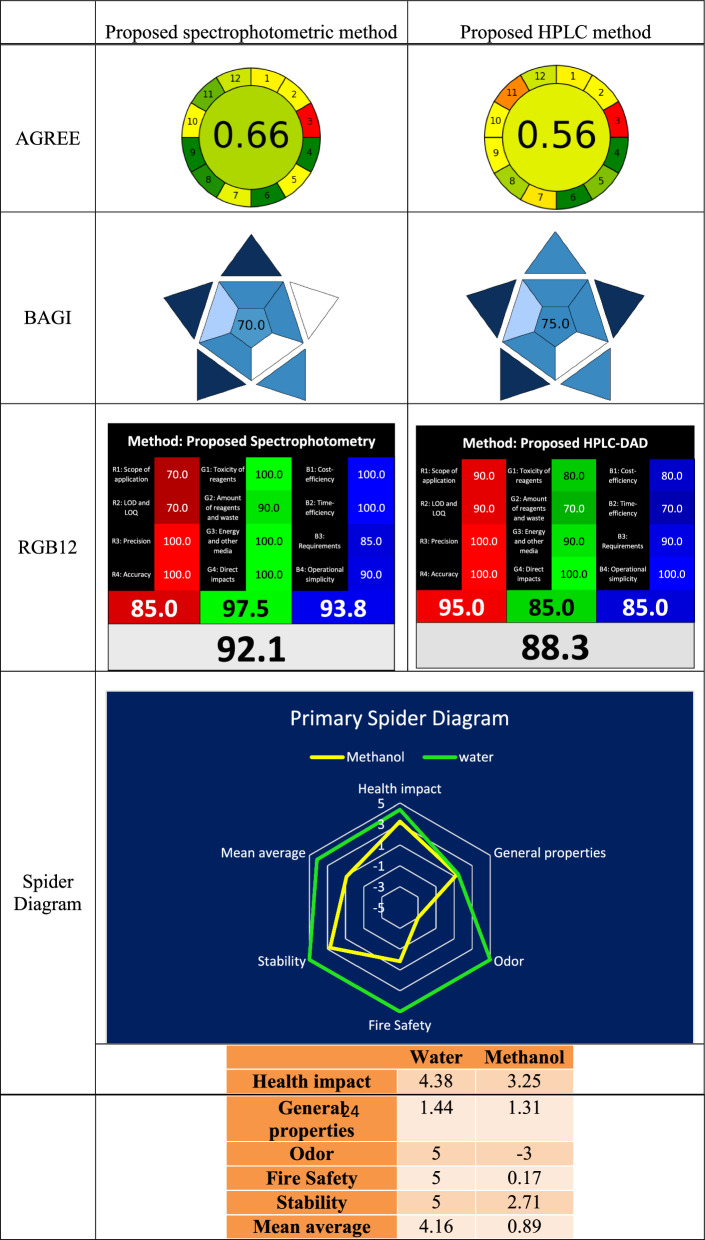
Comparison of the greenness, blueness and whiteness profiles of the proposed methods

#### Blueness assessment

GAC shouldn’t be the only criteria we ought to be taking into consideration before selecting a method. The method's applicability and practicality are equally significant. The red–green–blue (RGB) model, which integrates a method's ecological, analytical, and practical components and encompasses the "White Analytical Chemistry" concept (WAC), served as the model's inspiration for the color "blue" [44].

By taking into consideration ten important parameters: analysis type, analytes number, the instrumentation, the sample preparation requirements, the sample throughput, the number of analyzed samples per hour, the required chemicals and/or materials, automation degree, and the needed sample amount, BAGI enables a thorough assessment of the applicability or blueness of an analytical procedure. Each of these ten parameters is graded on a scale from 1 (worst) to 10 (best).

The composite BAGI score is computed as the geometric mean of the results for the ten distinct categories. An analytical technique with a higher BAGI score is considered more relevant, useful, and suitable for the purpose for which it is designed. With a high BAGI score of 70 and 75 for spectrophotometric and chromatographic methods, respectively, the proposed approaches demonstrated good blueness, Table 4. BAGI different scores between two methods is attributed to the small sample size needed for HPLC (10 µL) versus 3mL in spectrophotometry. Although BAGI review demonstrates that our approaches are very effective in terms of general functioning, hazard reduction, and time and cost savings, it is not able to provide a complete, thorough assessment of sustainability.

#### Whiteness assessment

The RGB12 model, introduced in 2021, marked the transition from green to white as a descriptor for analytical approaches, facilitated by an open-access, ready-to-use Excel spreadsheet. This WAC assessment tool is separated into three main color areas: red, green, and blue. The analytical efficiency is assessed in the red area using the validation criteria of sensitivity, accuracy, and precision. While the blue region evaluates performance using criteria including operational simplicity, cost-effectiveness, time efficiency, and lowest practical requirements, the green region gives GAC principles. The final score shows the whiteness percentage of the approach after calculating the arithmetic mean of the three regions following the evaluation of all RGB12 model parameters. Up to ten techniques can be evaluated and compared at once using the template. As our methods are the first ones for determination this synergistic combination, we used RGB12 model for comparison between them [45].

Every parameter is graded in relation to the best-performing approach. For example, the proposed HPLC method overwhelmed the spectrophotometric one in redness, 95 and 85, respectively, due to wider linearity range, lower LOD and LOQ beside higher robustness of the HPLC method, Table 4. On the other hand, as in AGREE, RGB12 greenness was higher using spectrophotometric techniques than the HPLC due to usage of greener solvent (water versus MeOH), lower waste volume (3 versus 6 mL), and lower energy consumption (due to shorter analysis time, 30 samples/h versus 10 samples/h), respectively, Table 4. In RGB12 blueness, the proposed spectrophotometric method gained higher score, 93.8, than the proposed HPLC one, 85. This was contradicted with BAGI results despite they pursuit the same goal: practicality of the method. We attributed this to the different evaluation elements in each of them beside self-assessment of RGB12 unlike automated BAGI. Nevertheless, the whiteness final score of both methods was higher than 85% which ensures sustainability of our methods, Table 4.

Looking to Table 4, RGB12 blueness score favors spectrophotometry over HPLC, contradicting the BAGI score due to different weighing factors used for each evaluation. RGB12 blueness evaluation depends on the average of four criteria’s: cost-efficiency, time-efficiency, requirements and operational simplicity giving the upper hand to the spectrophotometric method. While BAGI evaluation depends on ten criterions mentioned in Sect. "Blueness assessment" making it more powerful applicability evaluation tool beside its automation and HPLC was then bluer than the other methods.

### Spider diagram used to evaluate the solvent's greenness index

In our newly suggested spectrophotometric and HPLC methods, water and MeOH Greenness Index was assessed with spider diagram to evaluate the solvents used in each method, respectively. This tool depends on information obtained from safety data sheets (SDSs), which reveal details regarding a solvent's characteristics. A visual depiction of the overall sustainability degree for the chemicals utilized was created by combining five assessment criterion subcategories (health impact, fire safety, odor, overall characteristics, and stability) into a hierarchy spider diagram.

These criteria produced values that varied from − 5 to + 5. For each of the five previously listed subcategories, there are further spider charts that include extra information. Due to the fact that various chemical reagents do not offer all of the data required for the five aforementioned subgroups on a single SDS, the computation assigned a score of 0 to the missing data. The reliability of the greenness rating is demonstrated by these references. [39, 46, 47]

This spider method makes it possible to visually assess reagents, which simplifies and clarifies the thorough study of water and MeOH that were required for our procedures. Water has a positive average score of 4.16 on the major spider chart, Table 4, indicating that it is safe for both the environment and human health. This value is much higher than that of MeOH, 0.89. This explains why H_2_O is preferable than MeOH when giving similar sensitivities. The secondary spider diagrams in Fig. S4 a–d show the supporting information for the other scores. The percentage of relevant data and average scores for water and MeOH are illustrated in Table 4 of the Greenness Index.

The Spider Greenness Index technique offers a visual illustration of the solvent greenness comparison by elucidating the associated sub-points with each greenness requirement. However, this makes it challenging to adopt because it necessitates individual effort and requires reading through a large number of SDSs to find the most useful information, this makes it difficult to adopt.

### *Insilico* prediction of severity of DDIs

Drugs with cytochrome P450 (CYP) activity can act as substrates, inducers, or inhibitors of specific CYP enzymatic pathways, hence modifying the drugs’ metabolism that are supplied concurrently. Drugs that inhibit the CYP enzymatic pathway could produce medication toxicity due to a potential elevation in the plasma level of CYP substrates. Drugs that initiate a CYP enzymatic route can also cause drugs metabolized by the same process to have lower plasma concentration levels, which can lead to subtherapeutic levels of drug or unsatisfactory treatment outcomes. Most of the hepatic metabolism has been linked to the CYP enzymes coming from families 1, 2, or 3. Approximately 79% of the oxidation of these drugs was attributed to the most commonly engaged pathways, which included CYP2C9, CYP2D6, CYP2C19, and CYP3A4/5. Concurrent pharmaceutical therapy may result in drug-drug interactions. Therefore, comprehending the CYP system is vital to the safety of medications. [48]

DDIs mediated by CYP450 were assessed when the structures of the two compounds, TAD and MIR, were added to the Way2drug portal [49]**.** The likelihood of DDI at each individual enzyme level in the CYP450 class is indicated by the *IAP* data. The *IAP* is the Invariant Accuracy of Prediction which means the difference among the inactive probability "to be inactive" (Pi) and the active probability "to be active" (Pa) in DDIs mediated by CYP450. This value was calculated for the studied drugs. If *IAP* values are negative, this indicates that Pi > Pa as well as, quite likely, the drugs in question do not have any DDI at the cytochrome level. However, if *IAP* is positive and less than 0.7, there may be insufficient proof of DDI and it can be disregarded. But there is a considerable chance of DDI if *IAP* results are positive and more than 0.7.

Leave-one-out cross-validation (LOO CV) procedure is performed using the whole PASS training set for validation of prediction quality. Biological activity spectrum is predicted for each compound using the structure–activity relationships calculated from the data for all other compounds. The prediction result is compared with known experimental data for the studied compound. The procedure is repeated for all compounds from the PASS training set; then the average Invariant Accuracy of Prediction (IAP) values are calculated for each biological activity and for all biological activities.

**Fig. 8:**
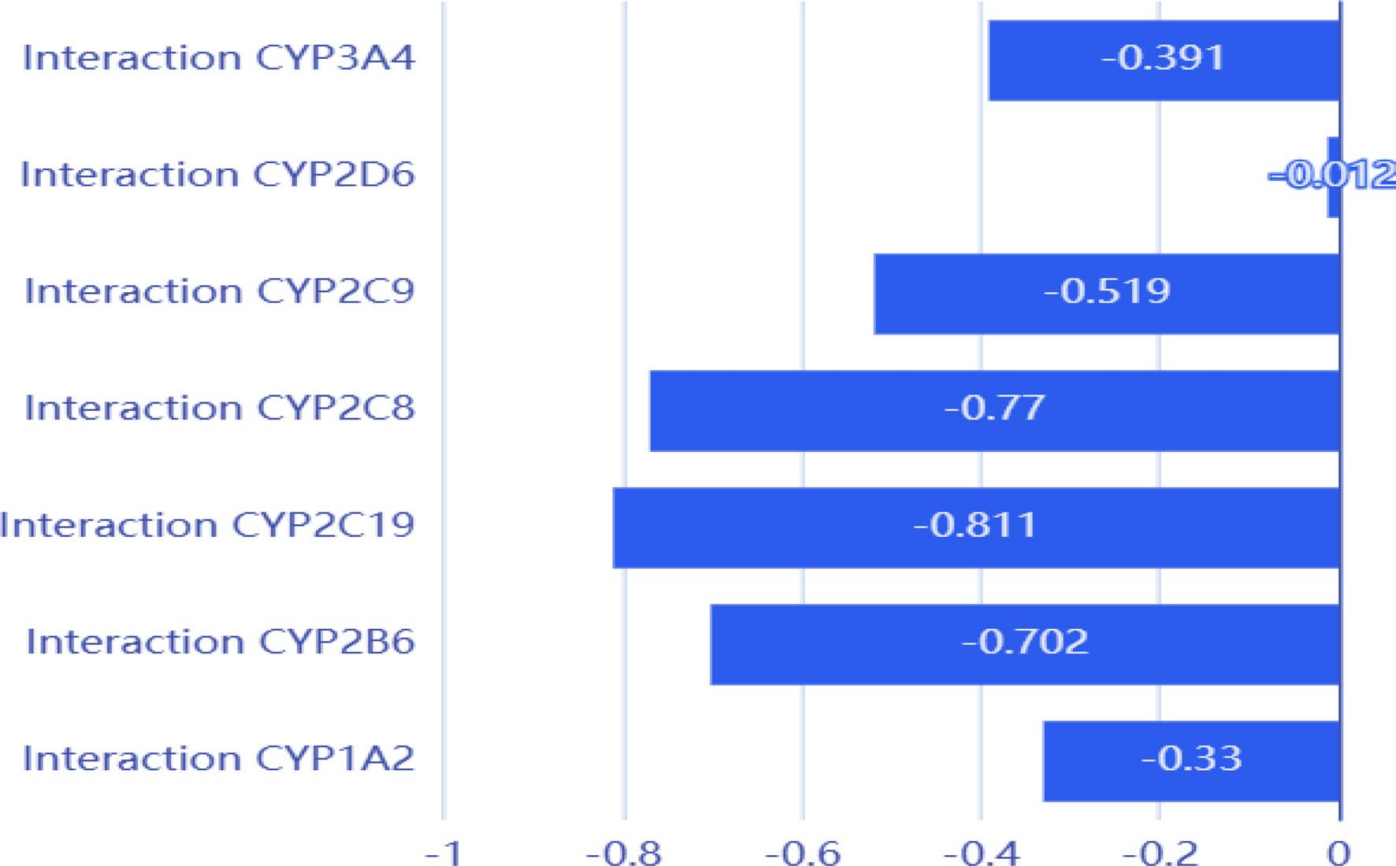
Diagram showing *IAP* values of DDIs between MIR and TAD at the level of the seven CYP450 enzymes

As shown in Fig. 8, there was no interaction between TAD and MIR at the seven CYP450 enzymes. The values of *IAP* were -0.391, -0.012, -0.519, -0.77, -0.811, -0.702, and -0.33 for CYP3A4, CYP2D6, CYP2C9, CYP2C8, CYP2C19, CYP2B6, and CYP1A2, respectively, Fig. 8. The ORCA system assessed the level of severity of DDIs and found that class 5, which denotes no interaction, was the case.

Moreover, the adverse effects due to concomitant administration were evaluated and all Pa values were lower than Pi ones. As activities for a given side effect are only deemed potential if Pa > Pi, this indicates no probability of interactions while coadministration of MIR and TAD, Table 5. [48]

**Table 5:** Possible DDI adverse events due to concomitant use of MIR and TAD

Activity	Pa	Pi
DDI arrhythmia	0,015	0,667
DDI bradycardia	0,068	0,368
DDI hypertension	0,075	0,259
DDI hypotension	0,079	0,412
DDI qt_interval_prolongation	0,018	0,664
DDI tachycardia	0,050	0,291

Therefore, MIR and TAD could be safely supplied together at the *insilico* level without compromising their pharmacokinetic properties. Our *insilico* approach, which guides but does not replace in vitro or in vivo research, can aid in prioritizing drug development efforts and initiates studies for polypills manufacturing possibilities.

## Conclusion

In conclusion, the developed analytical methods for the simultaneous determination of MIR and TAD in their combination therapy using spectrophotometry and HPLC offer highly reliable and environmentally sustainable solutions. Both methods were validated and demonstrated excellent performance in terms of precision, accuracy, and sensitivity, making them suitable for routine quality control applications. Moreover, the stability-indicating HPLC method successfully differentiated degradation products, confirming its suitability for stability studies. The assessment of these methods through green chemistry evaluation tools, such as AGREE, RGB12, and BAGI, confirms their eco-friendly nature, contributing to the advancement of sustainable analytical practices. Additionally, the use of artificial intelligence via an online tool to ensure the absence of DDIs further strengthens the safety profile of this combination therapy. Overall, this study provides a comprehensive, innovative approach to the analytical determination of MIR and TAD, ensuring their effective and safe use in clinical practice while promoting sustainability in pharmaceutical analysis.

## Supplementary Information

Supplementary material 1.

## Data Availability

Data are available upon request from the authors.
