# Supplementary material for: Cutting-edge assays for mirabegron and tadalafil combo therapy for benign prostatic hyperplasia; insilico kinetics approach; multi trait sustainability assessment
Source: BMC Chem. 2025 May 23;19(1):140. doi: 10.1186/s13065-025-01497-z (PMC12101028; doi:10.1186/s13065-025-01497-z)
Supplement: Supplementary file 1 — Supplementary material 1. [file 13065_2025_1497_MOESM1_ESM.docx]

(a)

(b)

**Fig. S1.** The coincident convoluted ratio spectra of (a) MIR 10 μg/mL alone& its mixture with TAD (1 μg/mL) using 1 μg/mL TAD as divisor, and (b) TAD 2 μg/mL alone & its mixture with MIR (20 μg/mL) using 10 μg/mL MIR as divisor.


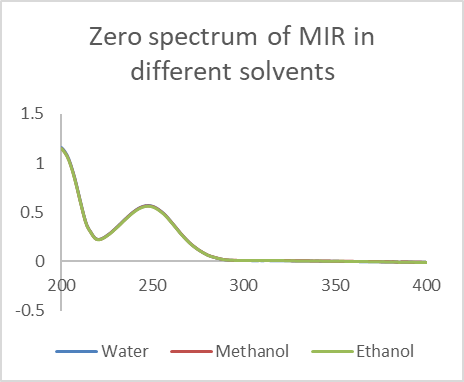


A


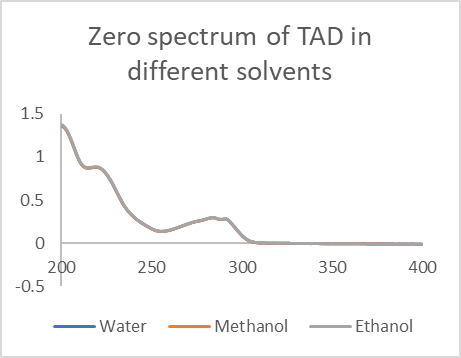


B

**Fig. S2. (A) overlay of MIR spectrum indifferent solvents (water, methanol, and ethanol). (B) overlay of TAD spectrum indifferent solvents (water, methanol, and ethanol)**


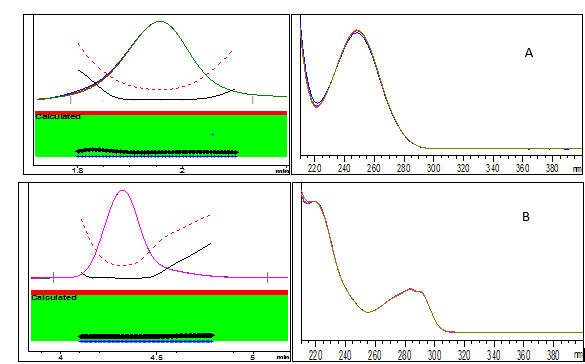


**Fig. S3.** Spectra and purity plot of A) MIR & B) TAD in HPLC.

**Fig. S4.** Secondary spider diagram for: A- Health impact, B- Stability, C- General properties, and D- Fire Safety for water and MeOH.

**Table S1. Different trials of mobile phase systems for optimization the HPLC method**

| **Trial** | **Condition** | **MIR Rt** | **MIR symmetry** | **Capacity factor MIR** | **TAD Rt** | **TAD symmetry** | **Capacity factor TAD** | **Resolution** | **Notes** |
| --- | --- | --- | --- | --- | --- | --- | --- | --- | --- |
| **1** | **Isocratic ACN: buffer 1:1** | **1.29** | **0.87** | **0.39** | **4.86** | **0.67** | **4.23** | **12.31** | **MIR & solvent front superimposed** |
| **2** | **Isocratic ACN: buffer 40:60** | **1.35** | **0.84** | **0.45** | **5.12** | **0.71** | **4.51** | **13** | **MIR & solvent front superimposed** |
| **3** | **Gradient ACN: buffer 80:20 for 3min then 20:80** | **1.19** | **0.92** | **0.28** | **4.12** | **0.84** | **3.43** | **10.1** | **MIR & solvent front superimposed & high noise in the baseline** |
| **4** | **Gradient ACN: buffer 40:60 for 3min then 80:20** | **1.89** | **0.75** | **1.03** | **5.23** | **0.82** | **4.62** | **11.51** | **High noise in the baseline** |
| **5** | **Isocratic MeOH: buffer 1:1** | **2.72** | **0.86** | **1.92** | **10.11** | **0.87** | **9.87** | **25.48** | **Long Rt for Mir** |
| **6** | **Isocratic MeOH: buffer 7:3** | **1.4** | **0.69** | **0.51** | **2.1** | **0.73** | **1.26** | **2.4** | **No clear solvent front** |
| **7** | **Isocratic MeOH: buffer 55:45** | **2.6** | **0.65** | **1.80** | **10.72** | **0.87** | **10.53** | **28** | **TAD took too long to elute** |
| **8** | **Gradient MeOH: buffer 40:60 for 2 min then 80:20** | **2.13** | **0.96** | **1.29** | **2.45** | **0.85** | **1.63** | **1.1** | **MIR and TAD peaks are very close together** |
| **9** | **Gradient MeOH: buffer 60:40 for 3 min then 80:20** | **1.95** | **0.86** | **1.10** | **4.3** | **0.78** | **3.62** | **8.09** | **Good separation** |

**Table S2.** Assay results for the determination of MIR and TAD in pure form using the proposed methods

| **%Recovery ^a^** | | | | | | | |
| --- | --- | --- | --- | --- | --- | --- | --- |
| **Analyte** | **MIR** | | | **TAD** | | | |
| **Method** | **^0^D/ff** | **R ^1^D** | **HPLC** | **^1^D** | **^0^D/ff** | **R ^1^D** | **HPLC** |
| **Mix 1 ^b^** | 98.00 | 100.20 | 98.11 | 99.10 | 101.90 | 100.10 | 101.99 |
| **Mix 2 ^c^** | 100.70 | 101.50 | 98.38 | 99.50 | 99.20 | 99.00 | 101.91 |
| **Mix 3 ^d^** | 99.40 | 101.80 | 99.28 | 99.00 | 98.20 | 99.70 | 100.20 |
| **Mean** | 99.37 | 101.17 | 98.59 | 99.20 | 99.77 | 99.60 | 101.37 |
| **SD** | 1.35 | 0.85 | 0.62 | 0.27 | 1.91 | 0.56 | 1.02 |
| **RSD%** | 1.36 | 0.84 | 0.62 | 0.27 | 1.92 | 0.56 | 1.00 |
| **^a^ average of the three determinations.** | | | | | | | |
| **^b^ Mix 1 containing 20 MIR and 2 TAD µg/mL (the dosage form ratio)** | | | | | | | |
| **^c^ Mix 2 containing 10 MIR and 10 TAD µg/mL** | | | | | | | |
| **^d^ Mix 3 containing 3 MIR and 15 TAD µg/mL** | | | | | | | |

**Table S3.** Evaluation of the robustness of the proposed HPLC method for the determination of MIR and TAD.

|  | MIR (250 nm) | | | TAD (225 nm) | | |
| --- | --- | --- | --- | --- | --- | --- |
| Parameters | **Mean % recovery ± SD ^a^** | **RSD% ^b^** | **t_R_ ± SD ^c^** | **Mean % recovery ± SD ^a^** | **RSD% ^b^** | **t_R_ ± SD ^c^** |
| Temperature (25 ± 2 ºC) | 98.59±0.12 | 0.12 | 4.30±0.09 | 101.07±0.04 | 0.04 | 1.94±0.03 |
| Wavelength of determination (selected λ ± 1 nm) | 99.64±0.05 | 0.05 |  | 101.37±0.30 | 0.30 |  |
| pH of water (7 ± 0.2 pH units) | 98.73±0.07 | 0.07 | 4.30±0.23 | 101.32±0.21 | 0.21 | 1.94±0.29 |
| Flow rate (1± 0.1 mL/min) | 99.43±0.03 | 0.03 | 4.30±0.79 | 100.71±0.11 | 0.11 | 1.94±0.91 |
| ^a^ Mean of percentage recovery of peak area of MIR & TAD of concentration 10 µg/mL at the studied parameters ± SD.  ^b^ Percentage relative standard deviation of % recovery of MIR & TAD at the studied parameters.  ^c^ Mean of retention time at the studied parameters ± SD. | | | | | | |

**Table S4.** Purity plots and UV spectra of MIR and TAD at different stress conditions using the proposed HPLC method

|  | MIR purity plot and UV spectrum | TAD purity plot and UV spectrum |
| --- | --- | --- |
| STD | 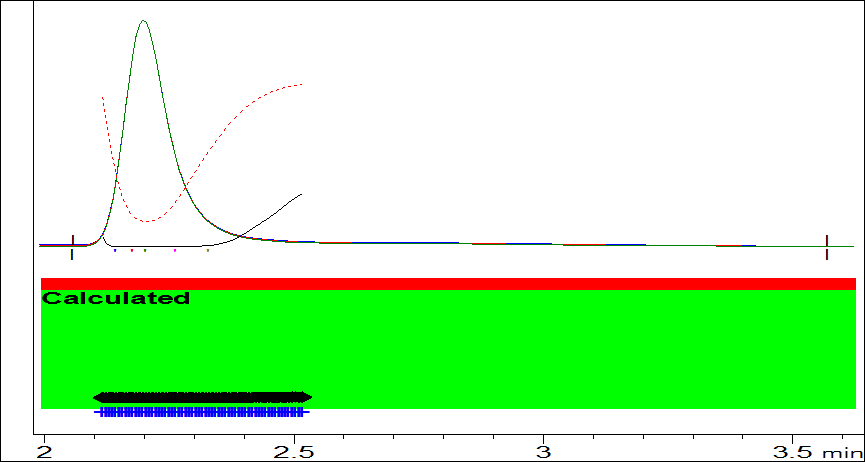 | 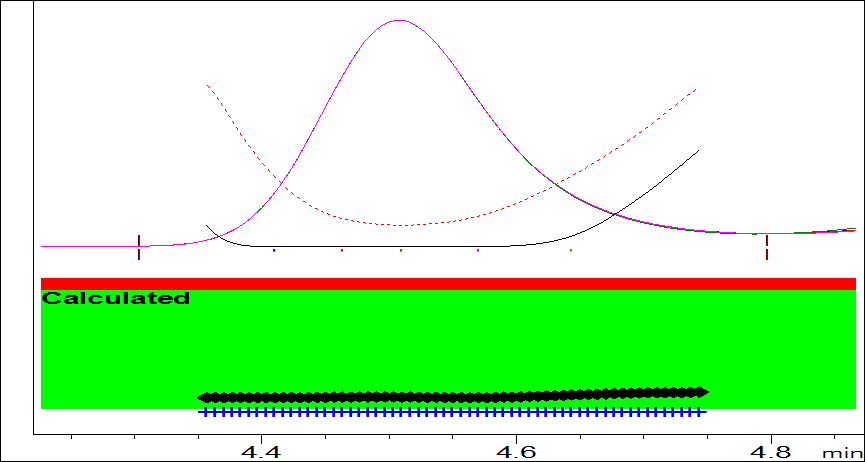 |
|  | 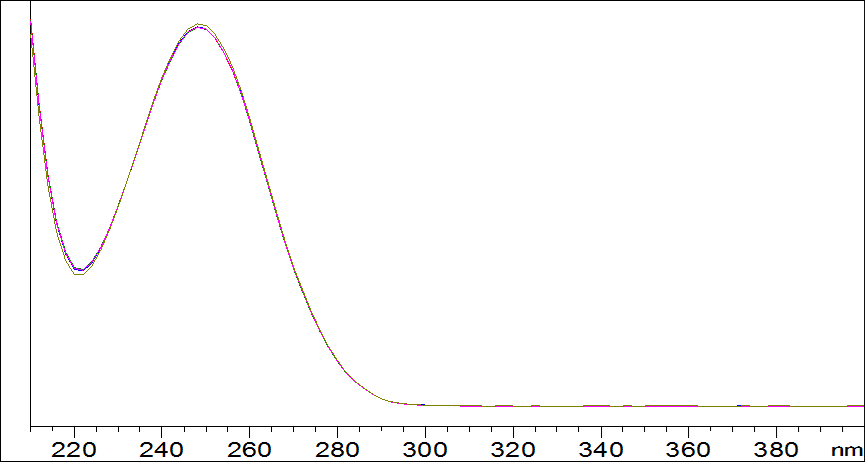 | 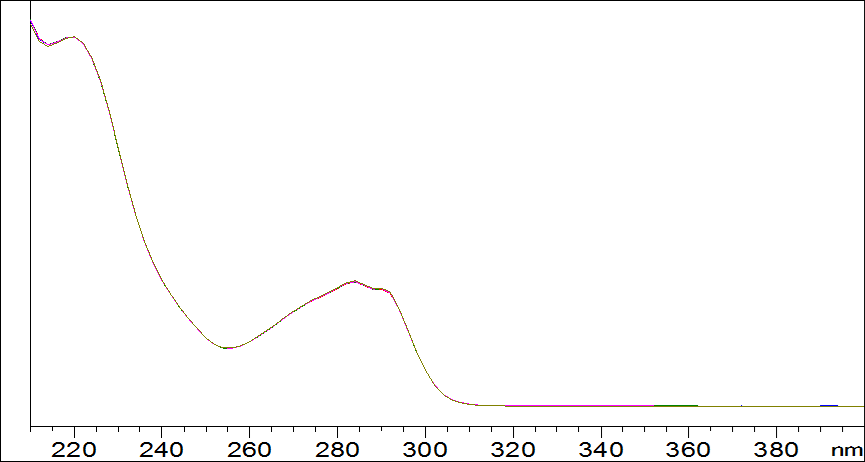 |
| NaOH |  | 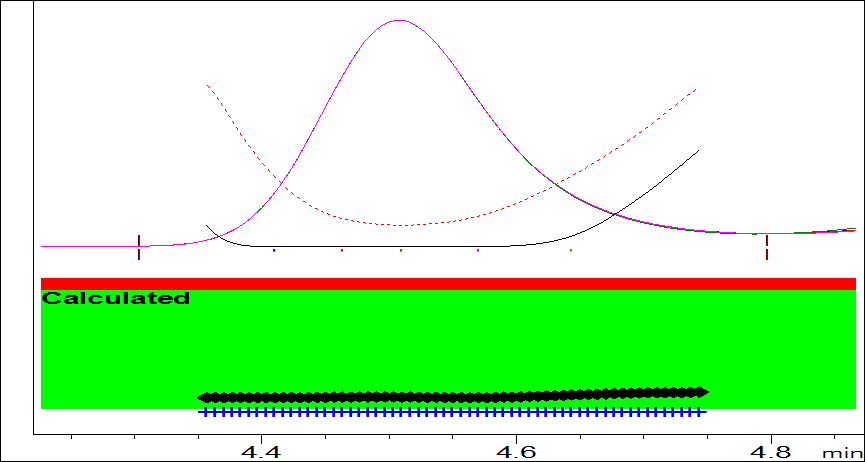 |
|  |  |  |
| HCl |  | 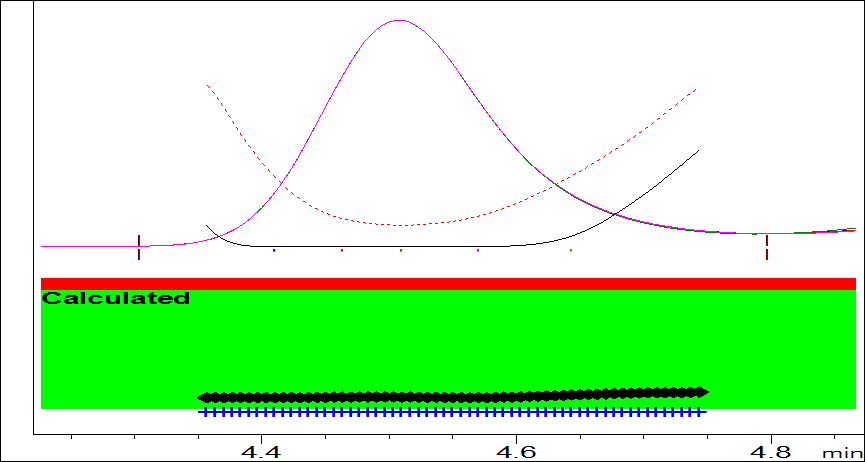 |
|  | 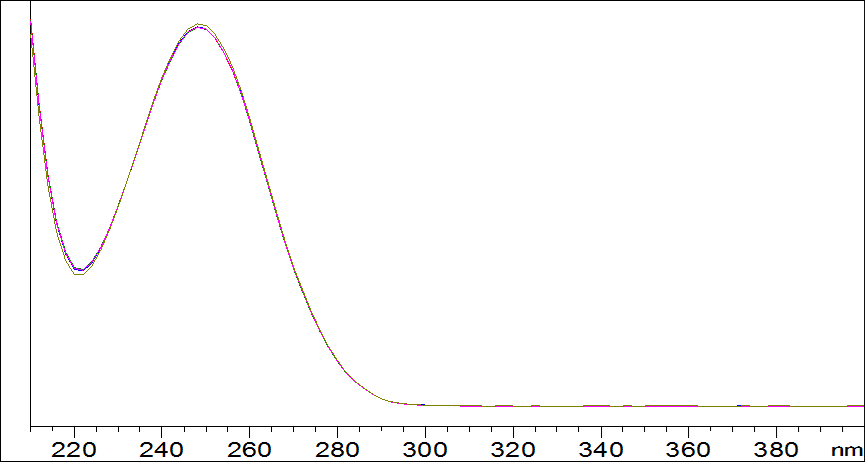 | 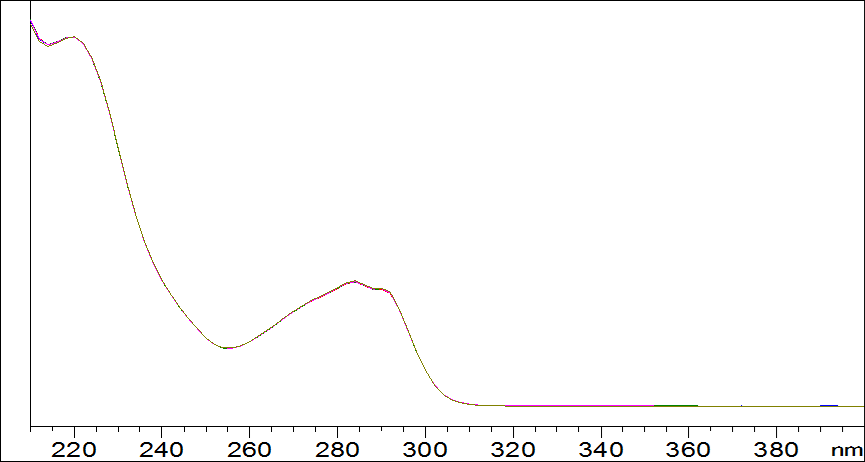 |
| H_2_O_2_ | 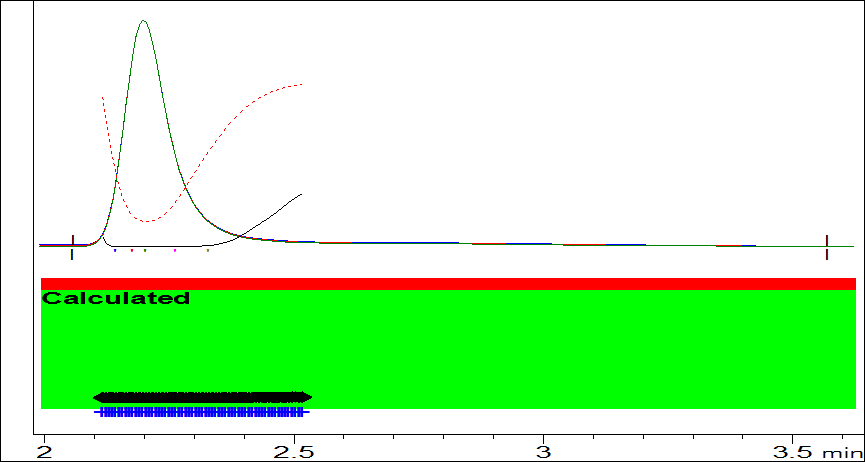 | 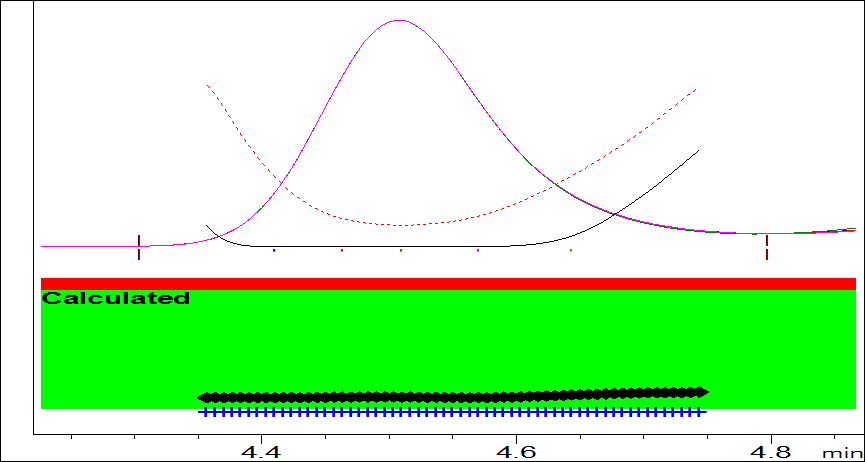 |
|  | 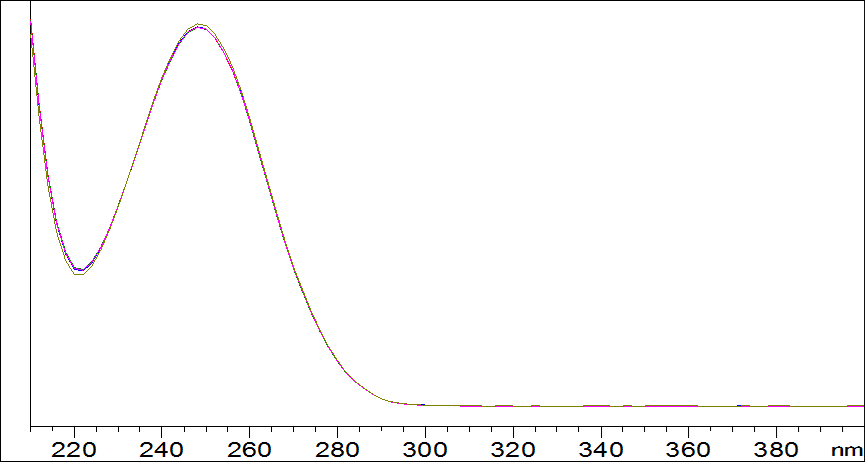 | 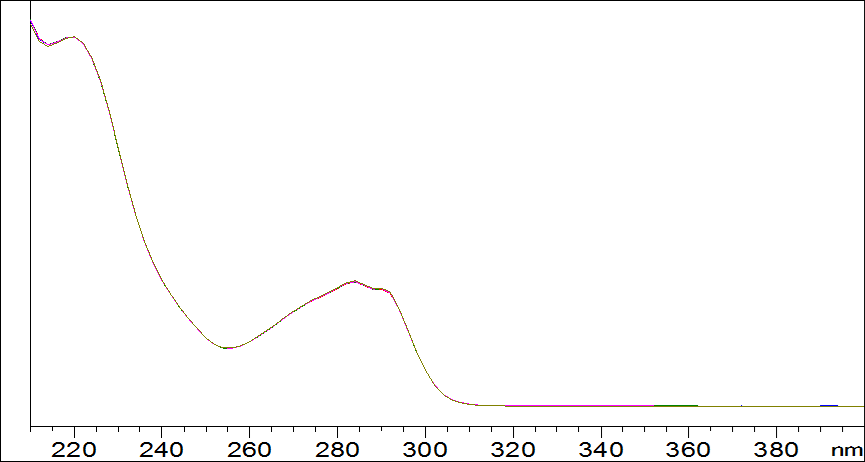 |
| H_2_O | 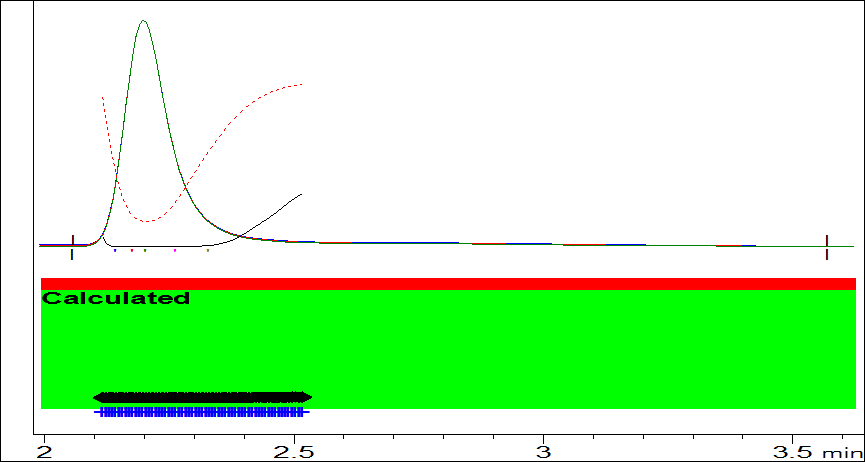 | 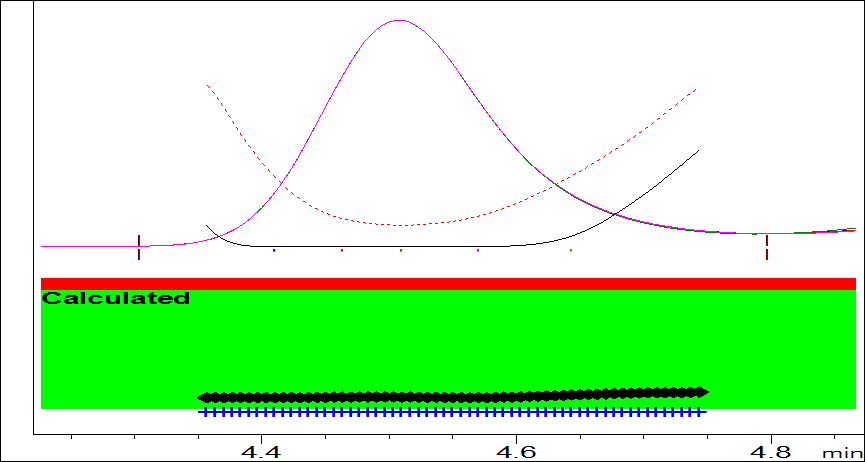 |
|  | 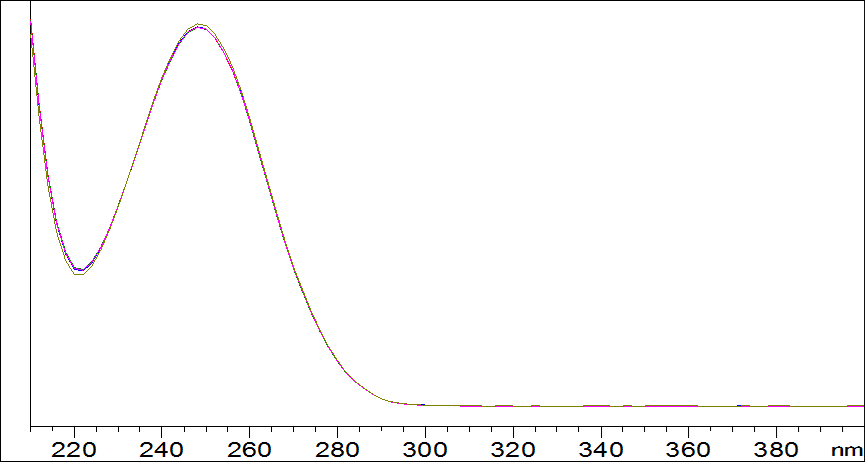 | 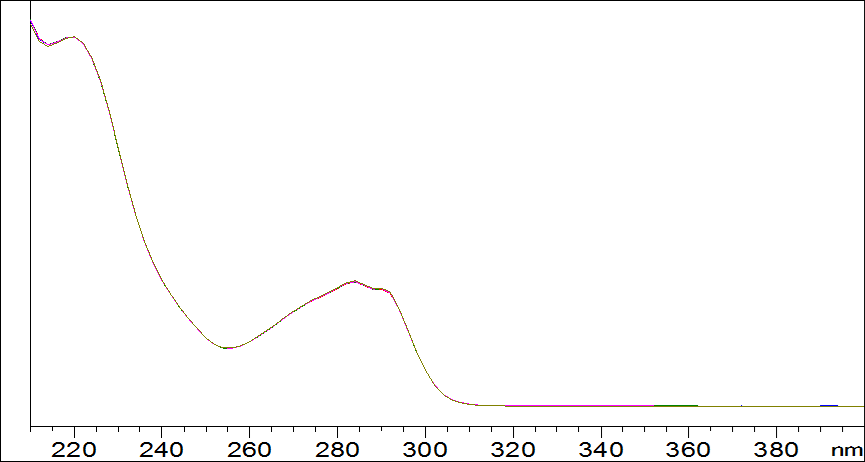 |
| Light | 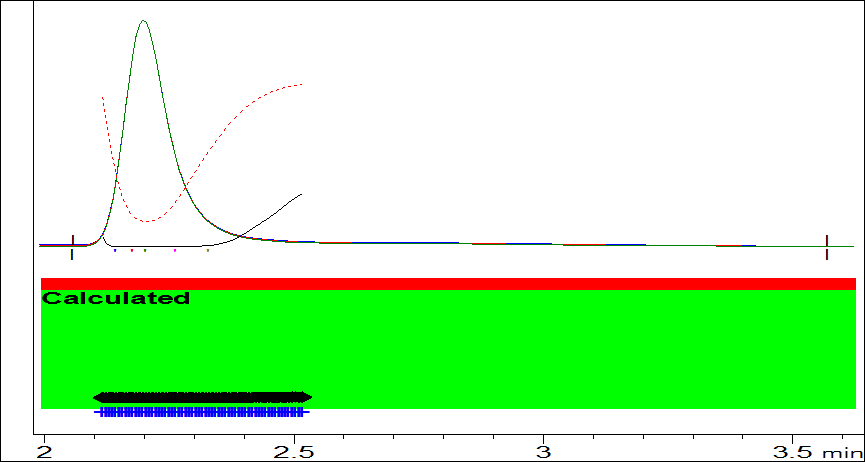 | 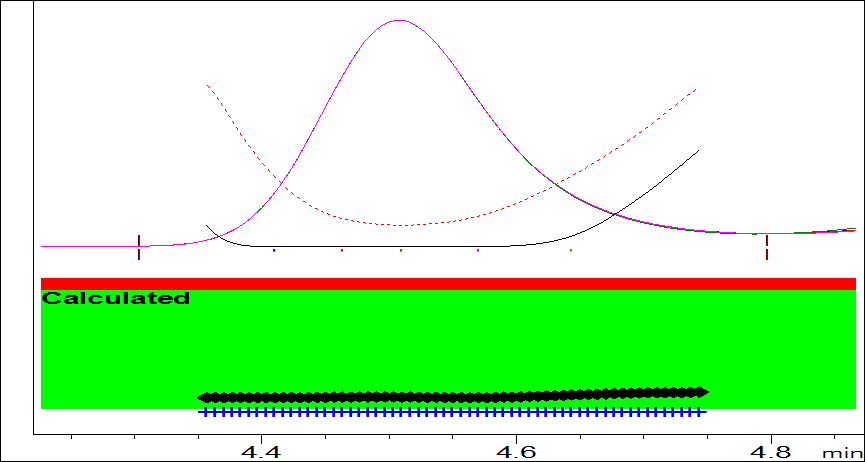 |
|  | 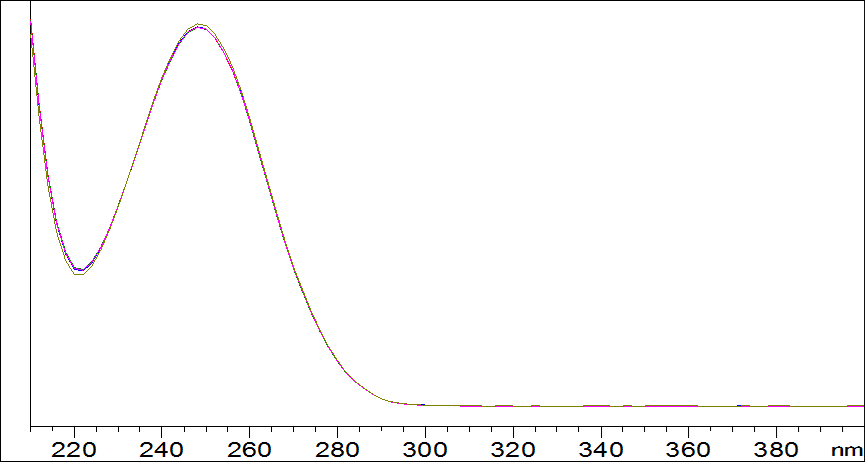 | 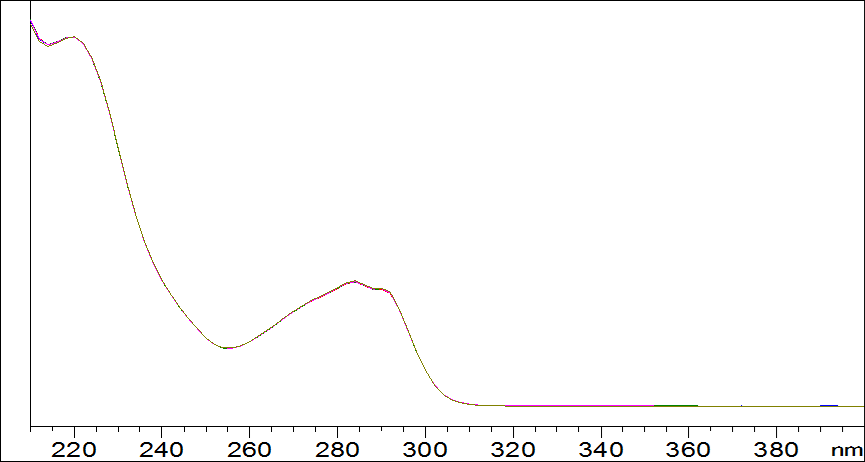 |
| Dry Heat | 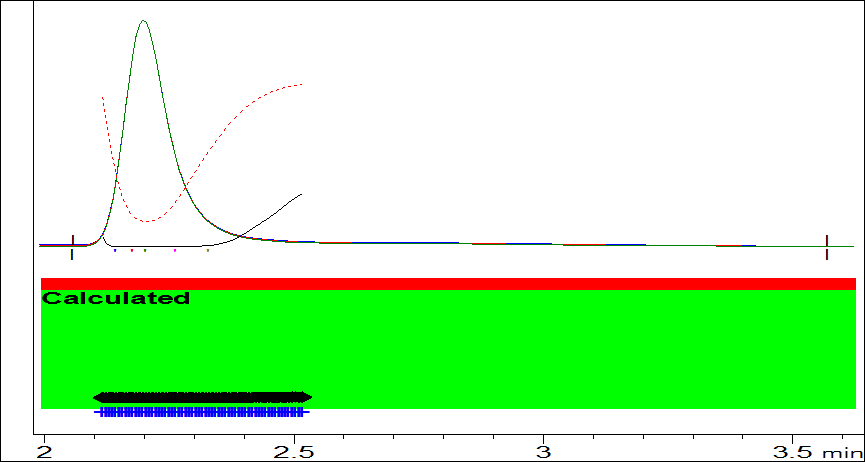 | 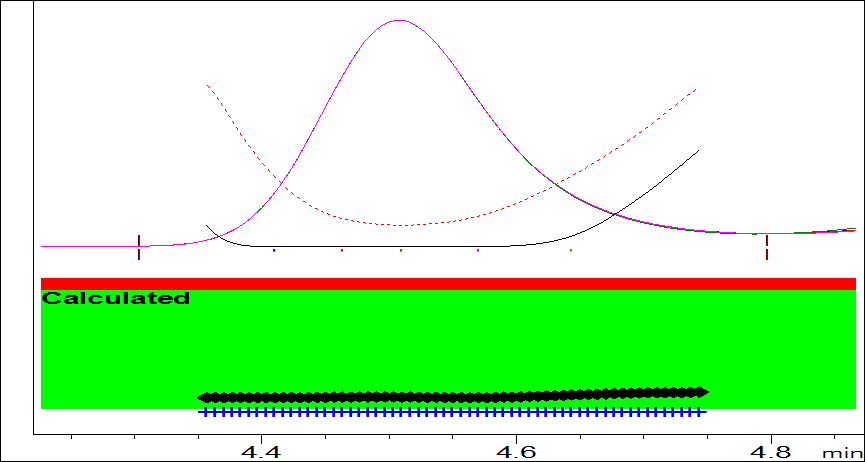 |
|  | 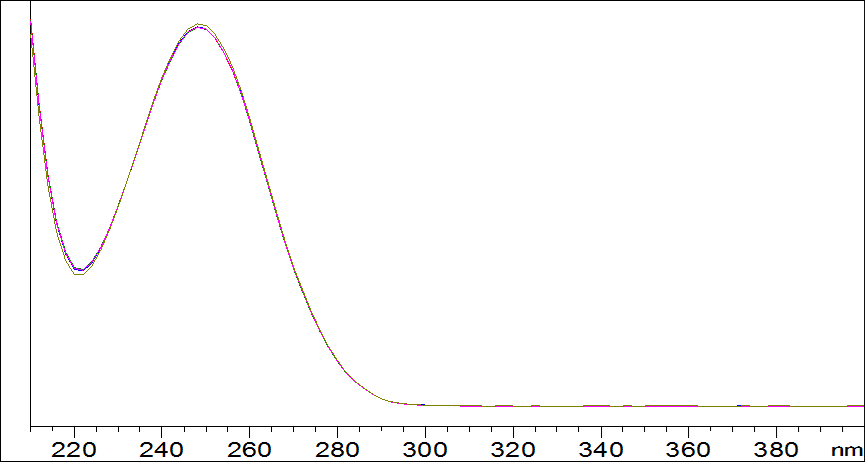 | 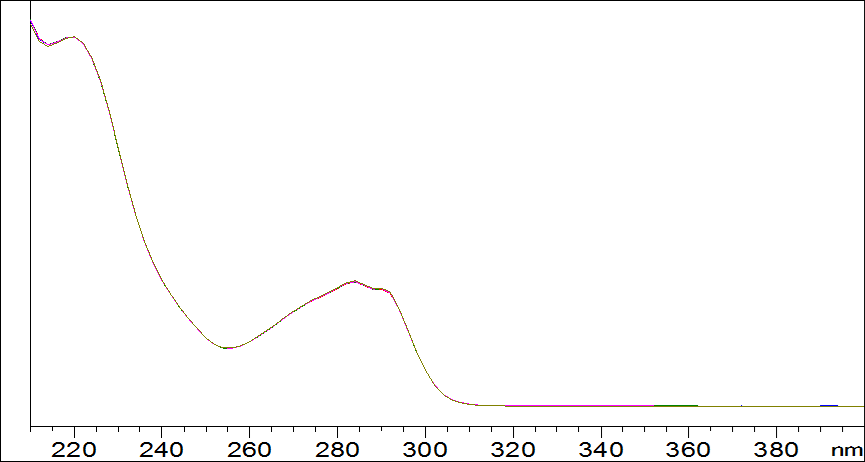 |
